# COVID-19 Infection and Vaccination Effects on Breast Implant Illness: A Case Report

**DOI:** 10.7759/cureus.69772

**Published:** 2024-09-20

**Authors:** Martin Bohac, Martina Chotárová, Dominika Mitevová, Alexander Mayer

**Affiliations:** 1 Plastic Surgery, Faculty of Medicine, Institute of Medical Biology, Genetics and Clinical Genetics, Comenius University Bratislava, Bratislava, SVK; 2 Plastic Surgery, University Hospital Bratislava, Bratislava, SVK; 3 Surgery, IV. Department of Surgery, Comenius University in Bratislava, Faculty of Medicine and University Hospital Bratislava, Bratislava, SVK

**Keywords:** autonomic dysregulation, breast augmentation, breast implant illness, capsulectomy, covid 19

## Abstract

Breast implant illness (BII) is a term used to describe a range of symptoms associated with silicone breast implants. This condition suggests that silicone may trigger symptoms in individuals who are immunologically predisposed, and the spectrum of symptoms may be linked to autonomic dysregulation in these patients. We present the case of a female patient in her mid-40s with a history of autoimmune thyroiditis who had not required prior therapy. She underwent breast augmentation and subsequently developed gradual difficulties. After recovering from a COVID-19 infection, her nonspecific symptoms, consistent with BII, worsened. These symptoms included chronic fatigue, hair loss, dry skin, petechiae, low-grade fever, and frequent urination. Additionally, she developed symptoms associated with long COVID. A subsequent Pfizer-BioNTech COVID-19 vaccination further exacerbated her symptoms and overall condition. Due to the broad spectrum of nonspecific symptoms, the patient underwent numerous screenings over an 18-month period, which were inconclusive. We hypothesize that the vaccination and previous infection had a synergistic effect on her ongoing BII symptoms, contributing to the worsening of her condition. An isolated right-sided seroma with left-sided lymphadenopathy appeared to be a side effect of the vaccine. Additionally, the patient developed a newly diagnosed allergy to polyethylene glycol and other allergic manifestations, such as chronic urticaria, which are consistent with autonomic nervous system dysregulation and long COVID. These symptoms resolved within three weeks of explantation with en bloc capsulectomy. It is noteworthy that the patient was unaware of BII until an MRI revealed a suspected intracapsular implant rupture on the right side, which led to the decision to remove the implants.

## Introduction

Breast implant illness (BII) is a recently coined term that has become increasingly recognized among both medical professionals and the general public. It encompasses a range of symptoms associated with silicone breast implants [1]. These symptoms are nonspecific and variable, including chronic fatigue syndrome, anxiety, depression, chest pain, palpitations, joint pain, hair loss, and knee pain, among others. Although some patients report symptom relief or complete resolution following implant removal, this improvement may be partly attributable to the nocebo effect [2,3]. The exact pathomechanism of BII is not yet fully understood, and no formal diagnosis exists for this condition. There are also no standardized guidelines for treating BII [4]. Ongoing studies are exploring various pathophysiological theories, including chronic T-lymphocyte activation in predisposed individuals, biofilm formation, and implant surface issues, but these theories often raise more questions than they answer. Biofilm, which involves resistant bacterial colonization of the implant surface, is linked to the production of autoantibodies or antibodies against specific bacteria, such as *Propionibacterium acnes *and *Staphylococcus epidermidis *[5,6].

Human adjuvant disease, first described by Myoshi in Japan in 1964 [7], refers to reactions to foreign materials like silicone implants in immunologically and psychosomatically predisposed individuals [8-10]. Such reactions can lead to the activation of autoimmune connective tissue diseases, including scleroderma, systemic lupus erythematosus, Sjögren’s syndrome, or rheumatoid arthritis [7]. It has been shown that silicone can migrate outside the capsule, leading to the formation of siliconomas, which have been histopathologically confirmed both inside and outside the capsule, presenting with site-specific symptoms [11-13].

There is limited scientific evidence regarding the impact of COVID-19 infection and vaccination on BII [14,15]. COVID-19 infection has been associated with the exacerbation or development of autoimmune diseases in some predisposed individuals [16,17]. Long COVID, chronic fatigue syndrome, fibromyalgia, chronic regional pain syndrome, and BII share a common factor: autoantibodies to the autonomic nervous system, collectively known as autoimmune autonomic dysfunction syndrome [18,19]. These autoantibodies target adrenergic receptors, muscarinic receptors, anti-endothelin receptor type A, anti-angiotensin receptor II type 1, and specifically beta1 adrenergic receptors, including immunoglobulin G (IgG) class autoantibodies against G protein-coupled receptors (GPCRs) [18-22].

Since the introduction of COVID-19 vaccines, side effects have ranged from minor issues like injection site tenderness and flu-like symptoms to more severe effects such as lymphadenopathy, fatigue syndrome, muscle and joint pain, nausea, vomiting, myocarditis, pulmonary embolism, stroke, and even sudden death [23-25]. Among patients with breast implants, adverse effects including lymphadenopathy, seroma, edema, redness, rash, fever, and capsular contracture (characterized by collagen fiber formation around the implant, leading to deformation and pain) have been reported following COVID-19 infection or vaccination [15,26]. Case reports have documented the onset of seroma around breast implants as early as two days and as late as 19 days after vaccination [26-30]. Additionally, a case was reported where a seroma developed two years after a TRAM flap and polypropylene mesh reconstruction, following a booster vaccination with an mRNA COVID-19 vaccine and subsequent COVID-19 infection [31]. Post-vaccination reactions involving dermal fillers have also been described [32]. Furthermore, one case report noted the sudden onset of capsular contracture type IV and lymphadenopathy at the vaccination site 21 days after receiving the second dose of the Pfizer-BioNTech vaccine [33]. These reactions suggest that immune-active cells around the implant or foreign material might be involved [33].

The potential synergistic effect of vaccination and prior COVID-19 infection in exacerbating BII symptoms remains a hypothesis, given the limited scientific data linking immune activation due to vaccination or infection directly to worsening BII symptoms. Further research is needed to understand how these immune triggers might interact with the pathophysiology of BII and potentially exacerbate existing symptoms.

## Case presentation

A female patient in her mid-40s, with no significant previous health problems, began experiencing symptoms following breast augmentation surgery. Her personal medical history included hyperthyroidism (Graves-Basedow disease) that spontaneously resolved towards the end of pregnancy, transitioning to a hypothyroid phase thereafter, which required no therapy. She also had a history of struma nodosa, a heart murmur attributed to a prolapsed mitral valve, MTHFR C677T heterozygote, MTHFR A1298C negative, PAI-1 homozygote 4G/4G, and negative for FV Leiden and FII G20210A. She was allergic only to penicillin and was lactose intolerant prior to the surgery.

The patient underwent breast augmentation using a periareolar approach with POLYTECH B-lite microthane 345cc implants, employing a dual-plane technique. The standard surgical protocol included a chirocaine pectoralis block, a Tegaderm nipple-areola complex (NAC) shield, and irrigation of the implant pocket with an antibiotic solution (clindamycin) diluted with betadine and saline, followed by a new pair of gloves (no talc). Monocryl 3/0 sutures were used for the skin and subcutaneous tissue, and Benelli sutures (Prolene 2/0) were used to secure the NAC position. Drains were removed 24 hours after surgery.

**Figure 1:**
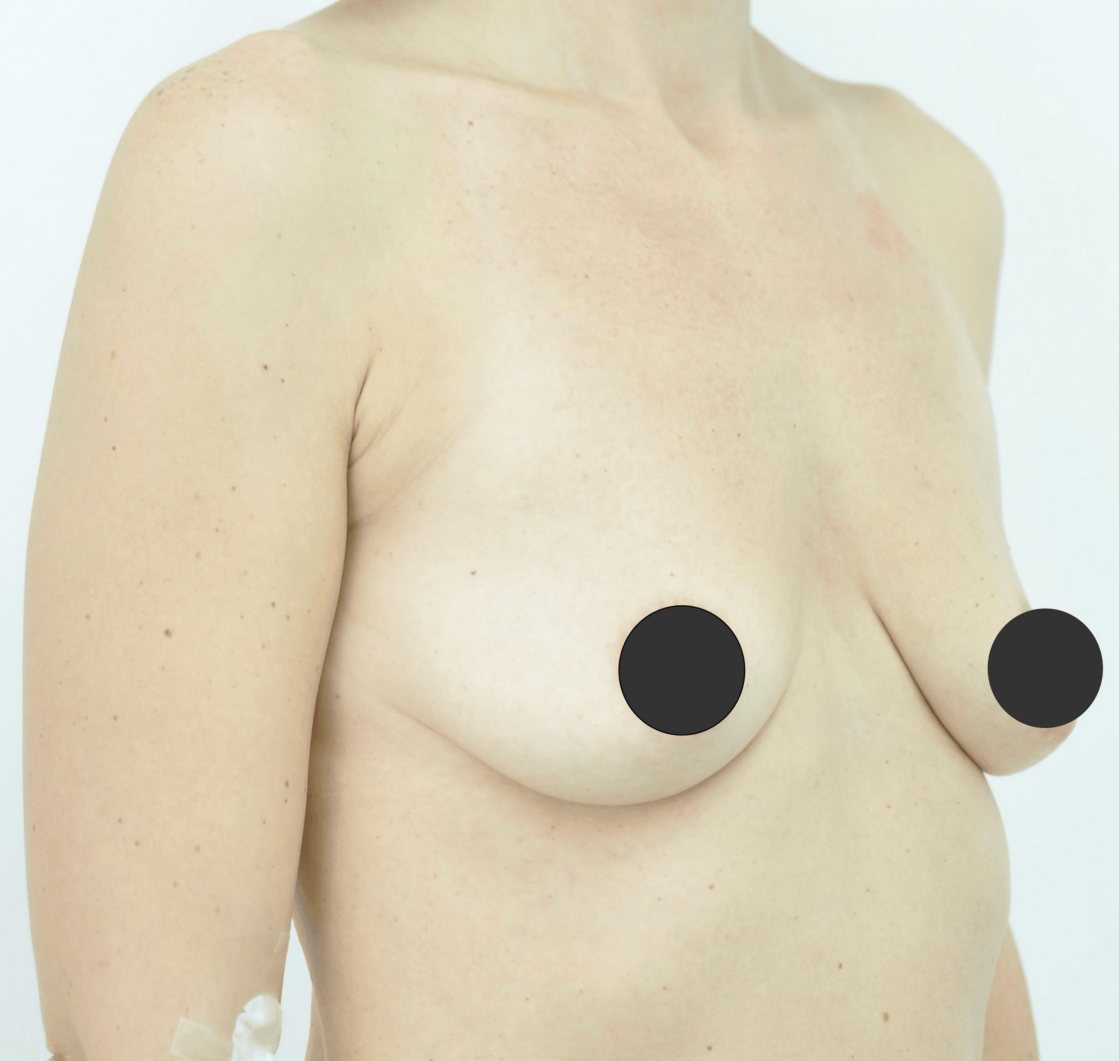
Presurgical image of the breast

**Figure 2:**
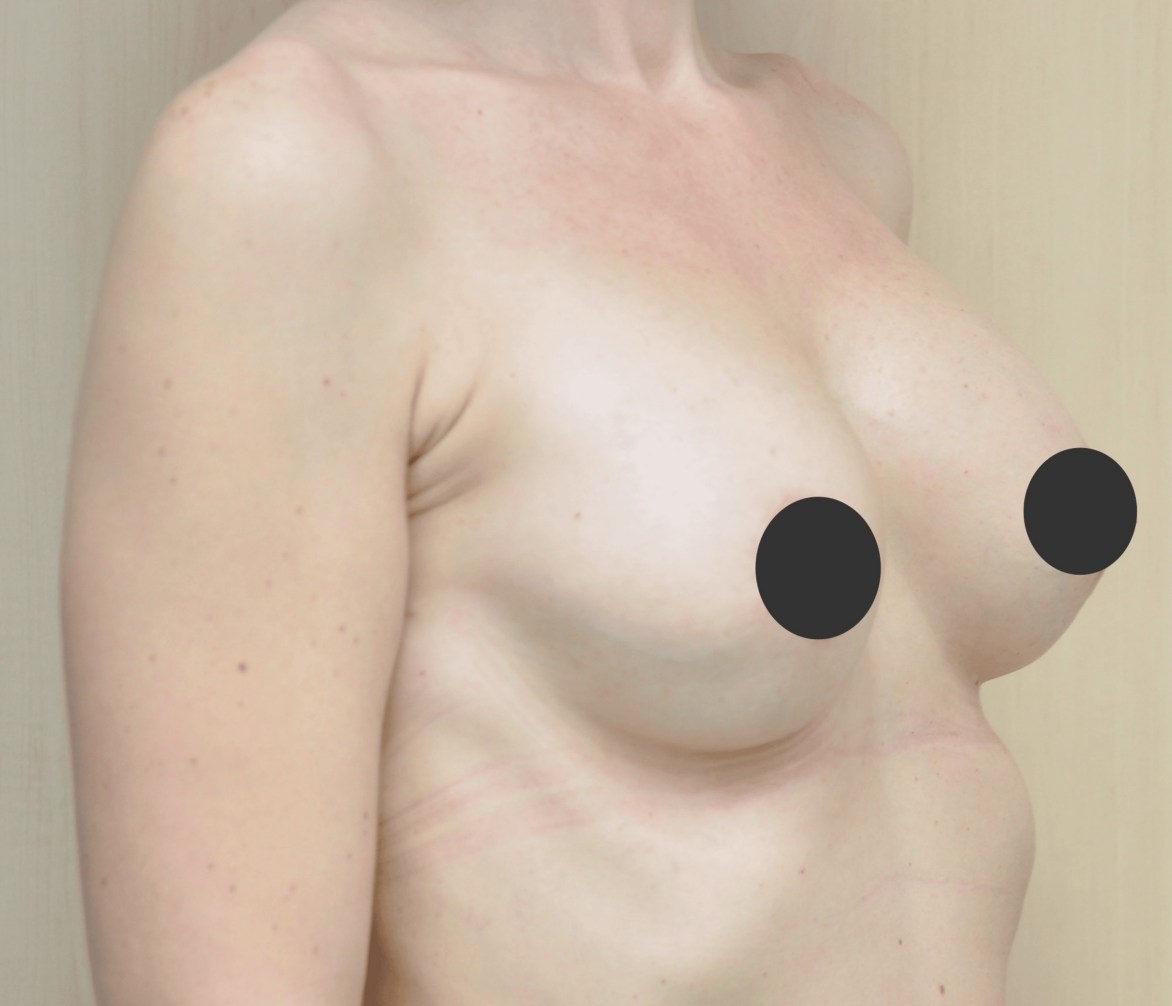
Postsurgical image of the breast

Postoperatively, the patient reported no complications, extraordinary pain, or other complaints. Antibiotics were administered preoperatively and continued for six days (clindamycin 300 mg every eight hours). The patient wore a compressive bra for six weeks postoperatively (Figure 1, Figure 2).

Several months after the augmentation, the patient began experiencing nonspecific symptoms, including dry skin, petechiae formation, facial paresthesia, insomnia, brain fog, and stabbing abdominal pain, among many others listed in Table 1 below.

**Table 1:** Symptoms experienced by the patient

Symptoms
Congested nose
Skin rash
Pruritus
Hair loss
Breast tenderness/pain
Chronic fatigue
Anxiety
Paresthesia of the face/limbs (cold limbs)
Stabbing abdominal pain
Menstrual cycle irregularities
Trouble with breathing
Macular skin lesions
Petechiae
Headache
Chest pain and persistent tachycardia (110-120/min)
Insomnia
Memory problems
Pain at the tip of the nose
Frequent night urination
Subfertility

Five months after the surgery, the patient contracted COVID-19 but managed to recover without hospitalization. Her symptoms included fever, weakness, headache, muscle and joint pain, and loss of smell. Following the COVID-19 infection, the symptoms listed in Table 1 worsened and further deteriorated after receiving two doses of the Comirnaty vaccine, administered six and seven months postoperatively, four weeks apart in the left arm. After the first dose, she developed migratory urticaria that resolved spontaneously within 24 hours. Despite prophylactic antihistamine therapy (levocetirizine), the second dose triggered pre-syncope lasting 10 days, hypertension unresponsive to medication, and a severe herpes simplex eruption extending from the upper lip to the nose and cheek.

During this period, the patient underwent several diagnostic tests, including a chest X-ray, spirometry, and a cardiological examination, due to persistent shortness of breath, chest pressure, increasing chest pain, and a new onset of paresthesia in the upper extremities. However, no significant findings were identified (Table 2). The patient’s condition was diagnosed as long COVID by a pulmonologist and an immunologist. She began breathing exercises and a therapeutic rehabilitation program, which provided minimal symptom relief.

**Table 2:** List of examinations and findings in chronological order

Examination	Time from augmentation	Finding	Blood test results	Histopathology	Microbiological examination
Chest X-ray	6 months	No finding (Figure 3)	Normal		
Spirometry	6 months	No finding			
Electrocardiography Holter, ECHO	6 months	No findings except for the mitral regurgitation, which the patient had already before the augmentation			
Gastrofibroscopy	6 months	Gastric cardia insufficiency dyspeptic syndrome		Mild chronic inactive gastritis; *Helicobacter pylori *immunohistochemistry: negative	
Ultrasonography of the abdomen	6 months	No finding	Normal		
Polysorbate allergy diagnosis	7 months	Confirmed allergy			
MRI of the cervical and thoracic spine	7 months	No significant finding			
Ultrasonography of the abdomen	9 months	Polyp in gallbladder	Normal except hyperbilirubinemia		
MRI of the cervical spine	9 months	Suspected meningioma in spinal canal C1 (Figure 4)			
Complete immunological examination	10 months		No significant finding, elevated level of histamine, thrombocytosis, low vitamin D levels, slightly elevated ASLO immunological finding – specific and nonspecific immunology panel was within the normal levels, borderline elevation of CD3+, CD3+HLADR+, complement levels within the normal range, lupus inhibitor in low titter 10/20, antiphospholipid antibody – negative hypersideremia – ferritin within normal range		
MRI of the breasts	11 months	No fluid around the implants			
Gynecological examination	13 months	Miscarriage on the seventh week			
MRI of the breasts	14 months	Fluid collection on the right side with a suspect of intracapsular implant rupture on the right side; no axillary nodes (Figure 5)			
Gynecological examination	14 months	No finding	Hormonal profile completely normal; decreased iron levels		
Mammography	16 months	Lymphadenopathy of one lymph node on the left side			
Ultrasonography	16 months	Fluid effusion around the right implant of approximately 20 ml, lymphadenopathy of two lymph nodes on the left side			
Explantation surgery	18 months	No implant rupture	Normal	Chronic inflammation in the capsule along with the deposits of silicone in the peri-implant capsule	Negative

**Figure 3:**
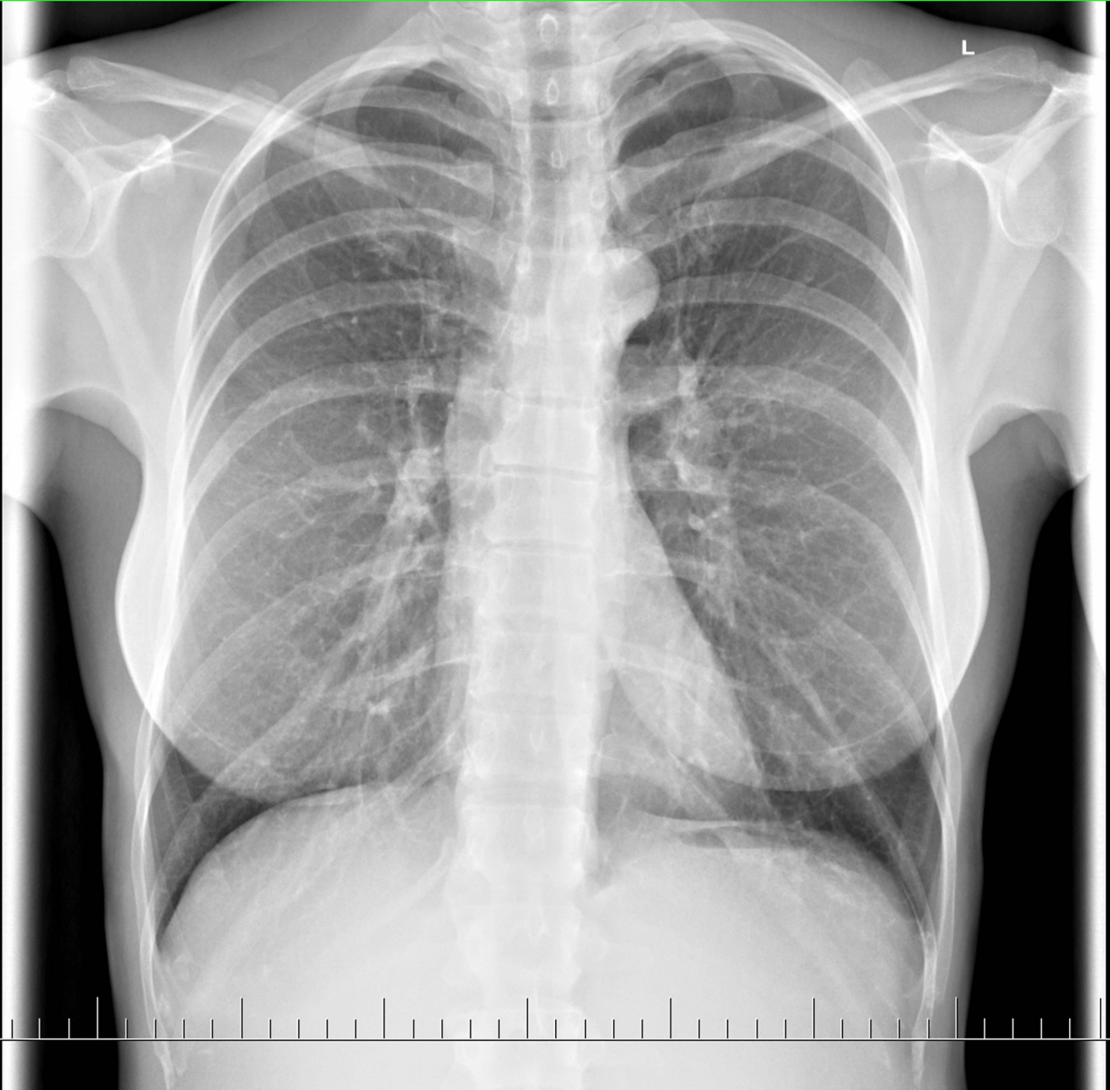
Chest X-ray after six months Chest X-ray showing no significant findings.

**Figure 4:**
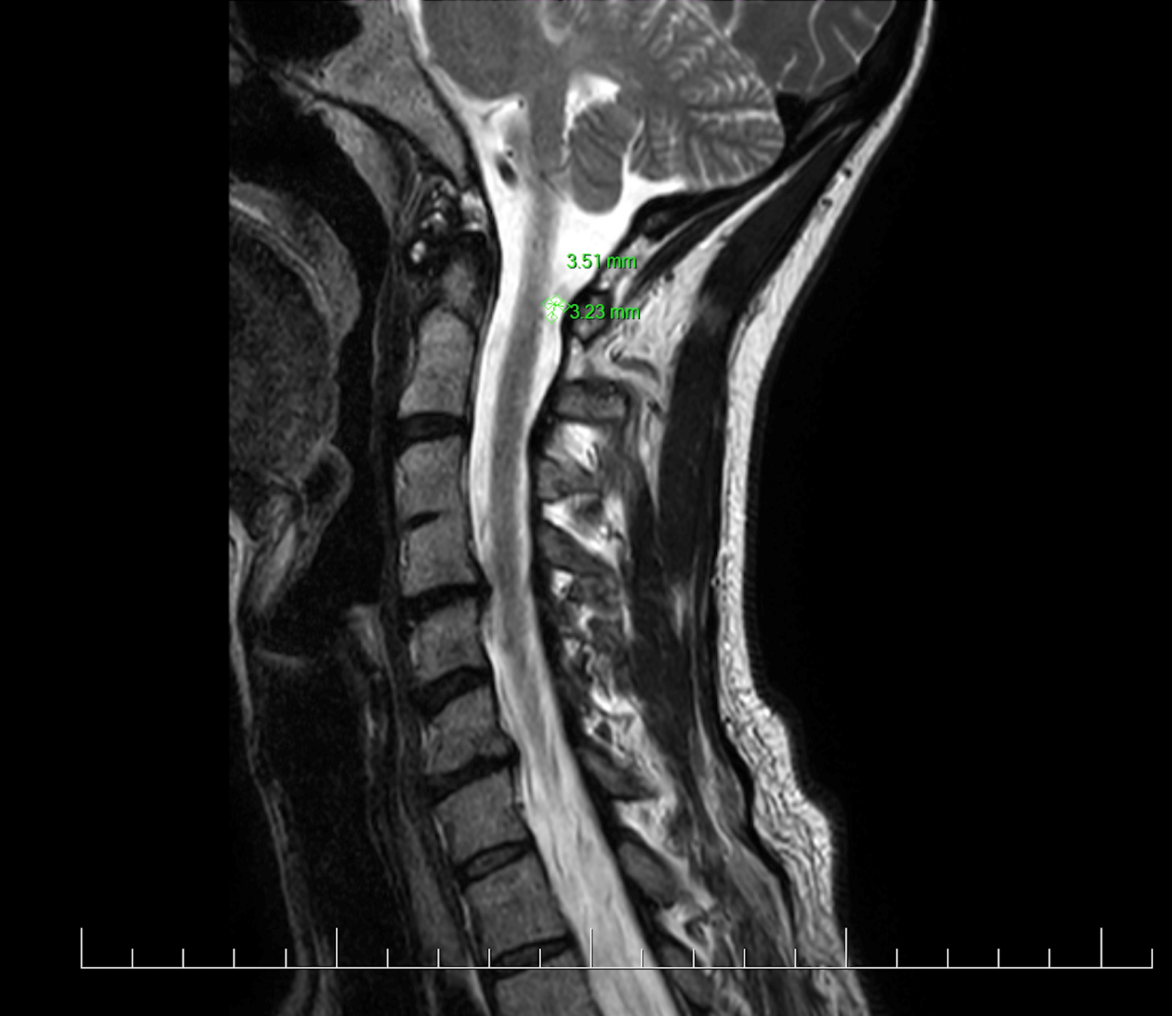
MRI of cervical spine nine months after augmentation MRI of the cervical spine showing a suspected meningioma in the spinal canal at C1 (green markings).

**Figure 5:**
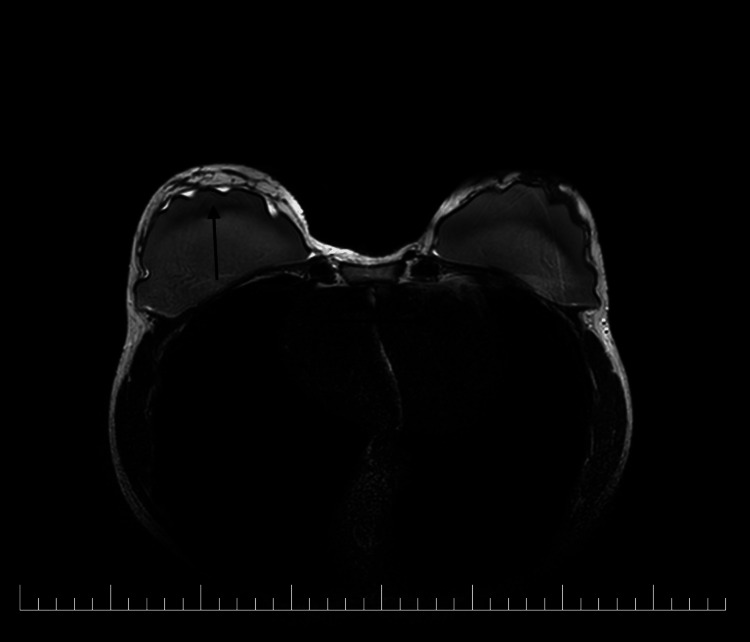
MRI of the breasts 14 months post-augmentation MRI of the breasts showing fluid collection on the right side, with suspicion of intracapsular implant rupture, as indicated by the black arrow. There is a suspected subcapsular rupture of the right implant, particularly in the upper quadrant, with fluid accumulation between the pectoral muscle and the upper edge of the implant.

The patient repeatedly visited the emergency department for stabbing abdominal pain. An ultrasound performed nine months post-augmentation revealed a gallbladder polyp and mild hyperbilirubinemia, leading to a diagnosis of biliary colic. She had no history of dietary errors and had not experienced such issues before. An earlier ultrasound (six months post-augmentation) and gastroscopy showed normal results.

Seven months post-surgery, the patient was diagnosed with allergies to polyethylene glycol (PEG), polysorbate, and aescin (which contains polysorbate). Following this diagnosis, the immunoallergist recommended carrying an EpiPen and antiallergic medications (desloratadine 2 × 5 mg, methylprednisolone 16 mg 2×, adrenaline 0.3 ml EpiPen, and ipratropium bromide). While POLYTECH stated that their implants do not contain PEG, the Comirnaty vaccine does.

Due to her ongoing symptoms, the patient underwent additional examinations, as detailed chronologically in Table 2. An MRI of the breast identified 20 ml of fluid around the right implant and suspected an intracapsular rupture (Figure 5). Based on these results, the patient opted for the removal of the silicone implants.

**Figure 6:**
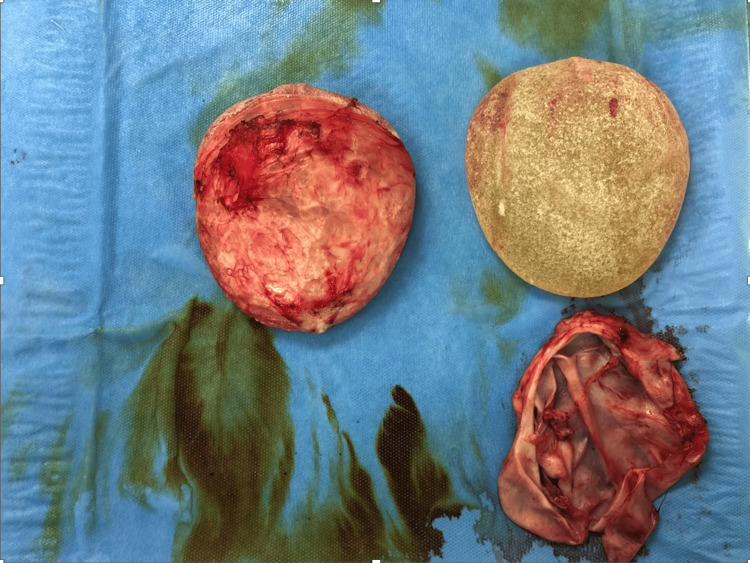
En bloc capsulectomy with removal of implants Image showing the en bloc capsulectomy procedure with the removal of breast implants and the surrounding capsule. No implant rupture was identified during the explantation, and less than 1 ml of fluid was observed around the right implant at the time of the procedure.

Intraoperatively, no macroscopic rupture of the implant was observed, and the capsules were found to be thin, as shown in Figure 6. Samples for intracapsular cultures and histological analysis were collected during the procedure. Postoperatively, the cultures returned negative results, while histological analysis revealed silicone deposits in the capsule along with chronic mild inflammation. Drains were removed 24 hours after the surgery. The healing process proceeded without complications, and the patient wore a compressive bra for six weeks.

The patient’s symptoms nearly all resolved following explantation. Insomnia disappeared immediately, and headaches and skin issues faded within three days. Hair loss and nasal apex pain subsided in under three weeks, and the menstrual cycle returned to normal. Some mild chest pain and cicatricial pain at the previous scar site persisted.

A three-month follow-up after the explantation surgery revealed symmetric breasts with no pain upon palpation, no inflammatory findings, and no axillary lymphadenopathy. The patient’s skin condition and chronic fatigue improved significantly. As of now, two years post-explantation, the patient remains free of BII symptoms and is doing well, with only mild cicatricial tenderness when weather conditions change.

## Discussion

Despite the periareolar approach generally carrying a higher risk of implant contamination compared to the submammary approach, no biofilm or capsular contracture was observed on the implant surfaces in this case. The capsule was thin, with minimal peri-implant fluid (less than 1 ml) around the right implant at the time of explantation. The surgical approach used adhered to current recommendations aimed at minimizing the risk of capsular contracture. Although macroscopic implant rupture was not confirmed, symptoms of BII can still develop even when the implant is intact [34]. Silicone particles were found in the peri-implant capsule along with chronic cellular inflammation, suggesting that the patient’s body was chronically irritated.

The patient also exhibited symptoms consistent with autoimmune autonomic dysfunction syndrome. Tanay et al. reported dysregulation of IgE alpha-1, beta-1 adrenergic receptors, and IgE muscarinic acetylcholine receptor type 1 in BII patients with allergic manifestations [21]. Elevated antibody levels were observed in both groups with BII symptoms, regardless of allergic manifestations, compared to healthy controls [21]. On the other hand, Tocut et al. observed decreased levels of IgG autoantibodies against GPCRs in patients with BII, which correlated with dysautonomic symptoms such as sleep disturbances, memory deficits, and depression [18,35]. Similarly, decreased antibody titers against autonomic nervous system receptors (including anti-α2AR, anti-β2AR, M1R, and M2R) were found in patients with palpitations and silicone implants compared to those without implants [36]. Despite the symptoms listed in Table 1 and the development of long COVID-19, standard immunological tests did not provide specific explanations for the patient’s condition.

The patient’s condition worsened significantly after COVID-19 infection and further deteriorated following vaccination, with the development of long COVID. Despite efforts to provide symptomatic relief, the patient’s symptoms only minimally improved and progressively worsened since the augmentation. The second MRI, conducted 14 months post-augmentation, revealed peri-implant effusion in the right breast and enlarged axillary lymph nodes on the left side, which were later confirmed on ultrasound 16 months after augmentation. Given that no breast imaging was performed immediately after augmentation, the findings on the left side might be coincidental or related to the vaccination or infection that occurred around six to seven months post-augmentation. The subjectivity of MRI descriptions should also be considered.

Explantation of the implants likely removed both the implants and the capsule, potentially halting the inflammatory response. Symptoms associated with BII resolved within three days to three weeks following explantation (typically within one month).

Autoimmune autonomic dysfunction syndrome has been linked with both BII and long COVID [19]. The exact mechanisms, especially regarding silicone implants and COVID-19 infection or vaccination, remain unclear and are currently under investigation [26-29,31,33]. Future research is needed to better understand how these immune triggers might interact with BII pathophysiology and potentially exacerbate symptoms.

Breast augmentation remains one of the most popular surgical procedures globally, with over 1.8 million performed annually, according to the 2023 ISAPS global survey. It is the second most common surgical procedure among women [37]. The incidence of requested breast revision surgeries has risen to 36% [38], often due to complications such as seroma, malposition, capsular contracture, or implant rupture. These issues may contribute to the development of BII in some patients [39,40]. The risk of complications increases with time, leading to a growing number of women seeking implant revision or removal. The 2023 ISAPS global survey reports approximately 106,000 implant removals [37]. Modern implants, particularly the fifth generation, are designed to be safer with a lower incidence of complications such as capsular contracture or rupture [41], although silicone leakage and rupture rates can vary.

Although BII is not yet officially recognized as a distinct diagnosis, it is crucial to address patient complaints and investigate potential causes. Due to the lack of precise guidelines for treating BII symptoms, comprehensive patient evaluation, including detailed anamnesis, physical examination, and additional tests, is essential. Current consensus suggests that the removal of implants, along with en bloc capsulectomy, is the most effective treatment for symptomatic patients [42]. However, en bloc capsulectomy remains controversial due to its invasive nature and potential risks, including removal of healthy tissue and complications such as asymmetrical deformities or pneumothorax, particularly in submuscular implant placements [43,44]. Therefore, en bloc or partial capsulectomy should generally be reserved for cases with confirmed breast implant-associated anaplastic large cell lymphoma (BIA-ALCL) [45,46].

## Conclusions

Although the silicone implant was not ruptured, histopathological examination revealed silicone particles within the capsule and chronic inflammatory cell infiltration. A small amount of fluid was detected around the right implant, and there were enlarged lymph nodes on the left side. The exact role of COVID-19 infection and vaccination in exacerbating the symptoms of BII remains uncertain. The decision to proceed with explantation was influenced by the suspected implant rupture, although this was not confirmed during the procedure. Nonetheless, the explantation resulted in a notable reduction of symptoms.

It is essential to take patient complaints related to BII seriously and conduct thorough investigations to rule out other possible diagnoses, such as BIA-ALCL and squamous cell carcinoma associated with breast implants. Comprehensive informed consent is crucial, ensuring that patients undergoing breast augmentation are fully aware of potential complications, including BII, especially if they have preexisting autoimmune conditions. Furthermore, it is important to recognize that COVID-19 infection or vaccination may exacerbate symptoms in predisposed individuals. However, additional research is necessary to develop definitive guidelines for managing patients affected by both BII and these external factors.
